# Viral Encephalopathy and Retinopathy in groupers (*Epinephelus* spp.) in southern Italy: a threat for wild endangered species?

**DOI:** 10.1186/1746-6148-9-20

**Published:** 2013-01-26

**Authors:** Niccolò Vendramin, Pierpaolo Patarnello, Anna Toffan, Valentina Panzarin, Elisabetta Cappellozza, Perla Tedesco, Antonio Terlizzi, Calogero Terregino, Giovanni Cattoli

**Affiliations:** 1 Istituto Zooprofilattico Sperimentale delle Venezie, viale dell’Università, 10-35020, Legnaro, Padova, Italy; 2 Current affiliation: EURL for fishdiseases, DTU VET, Hangovej 2, Aarhus, Denmark; 3 Dipartimento di Scienze e Tecnologie Biologiche ed Ambientali, Università del Salento, CoNISMa, Lecce, Italy; 4 Fish Patologist, Private Expert, Istituto Zooprofilattico Sperimentale delle Venezie, viale dell’Università, 10 -35020 Legnaro, Padova, Italy

**Keywords:** Viral Encephalopathy and Retinopathy, Betanodavirus, Neurological signs, Wild fish, *Epinephelus* spp.

## Abstract

**Background:**

Betanodaviruses are the causative agents of Viral Encephalopathy and Retinopathy (VER). To date, more than 50 species have proved to be susceptible and among them, those found in genus *Epinephelus* are highly represented. Clinical disease outbreaks are generally characterized by typical nervous signs and significant mortalities mainly associated with aquaculture activities, although some concerns for the impact of this infection in wild fish have been raised. In this study, the authors present the first documented report describing an outbreak of VER in wild species in the Mediterranean basin.

**Case presentation:**

In late summer - early winter 2011 (September-December), significant mortalities affecting wild Dusky grouper (*Epinephelus marginatus*), Golden grouper (*Epinephelus costae)* and European sea bass (*Dicentrarchus labrax*) were reported in the municipality of Santa Maria di Leuca (Northern Ionian Sea, Italy). The affected fish showed an abnormal swimming behavior and swollen abdomens. During this epizootic, five moribund fish showing clear neurological signs were captured and underwent laboratory investigations. Analytical results confirmed the diagnosis of VER in all the specimens. Genetic characterization classified all betanodavirus isolates as belonging to the RGNNV genotype, revealing a close genetic relationship with viral sequences obtained from diseased farmed fish reared in the same area in previous years.

**Conclusion:**

The close relationship of the viral sequences between the isolates collected in wild affected fish and those isolated during clinical disease outbreaks in farmed fish in the same area in previous years suggests a persistent circulation of betanodaviruses and transmission between wild and farmed stocks. Further investigations are necessary to assess the risk of viral transmission between wild and farmed fish populations, particularly in marine protected areas where endangered species are present.

## Background

Viral Encephalopathy and Retinopathy (VER) is a viral infectious disease affecting more than 50 marine fish species worldwide and it is considered one of the most important threats for mariculture in the Mediterranean.

Fish affected by VER generally show clear clinical signs such as anorexia, skin darkening, blindness and abnormal swimming behaviour. Typical histological lesions include cellular vacuolation, necrosis and neuronal degeneration in the central nervous system (CNS).

The aetiological agent of VER is comprised in the family *Nodaviridae*, genus *Betanodavirus*, and it is an icosahedral, non-enveloped viral particle of about 25 nm in diameter, with a genome made of two single-stranded positive-sense RNA molecules. The RNA1 (3.1 Kb) and the RNA2 (1.4 Kb) genetic segments encode the RNA-dependant RNA-polymerase (RdRp) and the coat protein respectively [1], while the RNA3 subgenomic transcript originating from the RNA1 molecule (0.4 Kb) is translated into protein B2, which is involved in viral intra-cellular replication mechanisms [2]. On the basis of the phylogenetic analysis of the T4 variable region within the RNA2 segment, five genotypes have been described to date: striped jack nervous necrosis virus (SJNNV), tiger puffer nervous necrosis virus (TPNNV), barfin flounder nervous necrosis virus (BFNNV), red-spotted grouper nervous necrosis virus (RGNNV) and turbot nervous necrosis virus (TNNV) [3,4]. Recently, betanodavirus reassortant strains possessing genome segments belonging to parental RGNNV and SJNNV have been also described [5-7].

The genus *Epinephelus* is considered highly susceptible to betanodavirus infection. Wild and farmed groupers have been frequently involved in natural outbreaks of the disease in Asian countries: i.e. mortality caused by VER has been described in *E. fuscoguttatus, E. akaara* in Japan, *E. septemfasciatus* in Japan and Korea, *E. coioides* in Philippines, *E. awooara* in Taiwan*, E. tauvina* in Malaysia, Philippines and Singapore*, E. moara* in Japan [8]*, E. lanceolatus* and *E. malabaricus* in Taiwan [9] and *E. marginatus* in Taiwan [10].

In the Mediterranean Sea, VER represents a major limiting factor for the development of *E. marginatus* rearing activity both for feeding consumption [11] and preservation programs [12]*,* and the virus has been sporadically detected in wild *E. aeneus*[13] and *E. costae*[5]. Several epidemiological surveys highlighted the presence of betanodavirus in farmed animals and wild asymptomatic fish stocks other than groupers in the Mediterranean basin [5,7,12-15]; thus underlying the widespread of this virus in this area. Although rumors of groupers mortality in several areas of the Mediterranean Basin, such as Sicily, Corsica, the Balearic Islands and other parts of the Spanish coast, Algeria, Tunisia and Greece, have circulated in the recent past, to the authors’ knowledge a description of the phenomenon, as well as the demonstration of the causative agents have never been clearly documented.

From an ecological and economic point of view, *Epinephelus spp*. is considered one of the most relevant fish genus for the Mediterranean sea; moreover, some grouper species, i.e. *E. marginatus*, are considered endangered [16], and their survival is safeguarded in marine protected areas (MPA) where angling and scuba diving are strictly regulated.

In this paper an outbreak of severe disease and mortality associated to betanodavirus infection in wild fish, including groupers, is described.

### Case presentation

The outbreak started in late September 2011 and lasted until the beginning of December 2011, and was characterized by extraordinary climatic conditions. At the end of summer 2011, unusual high temperatures of the entire water column were recorded for more than 30 days. In the period between September-November 2011 the mean water temperature varied from 25.99°C-17.57°C at 5 meters depth to 21.82°C-17.21°C at 35 meters depth (authors’ unpublished data). Most likely, the uniform high temperature in all the water column was caused by a prolonged elevated surface pressure associated with the absence of meteorological disturbances that deepened the thermocline.

**Figure 1:**
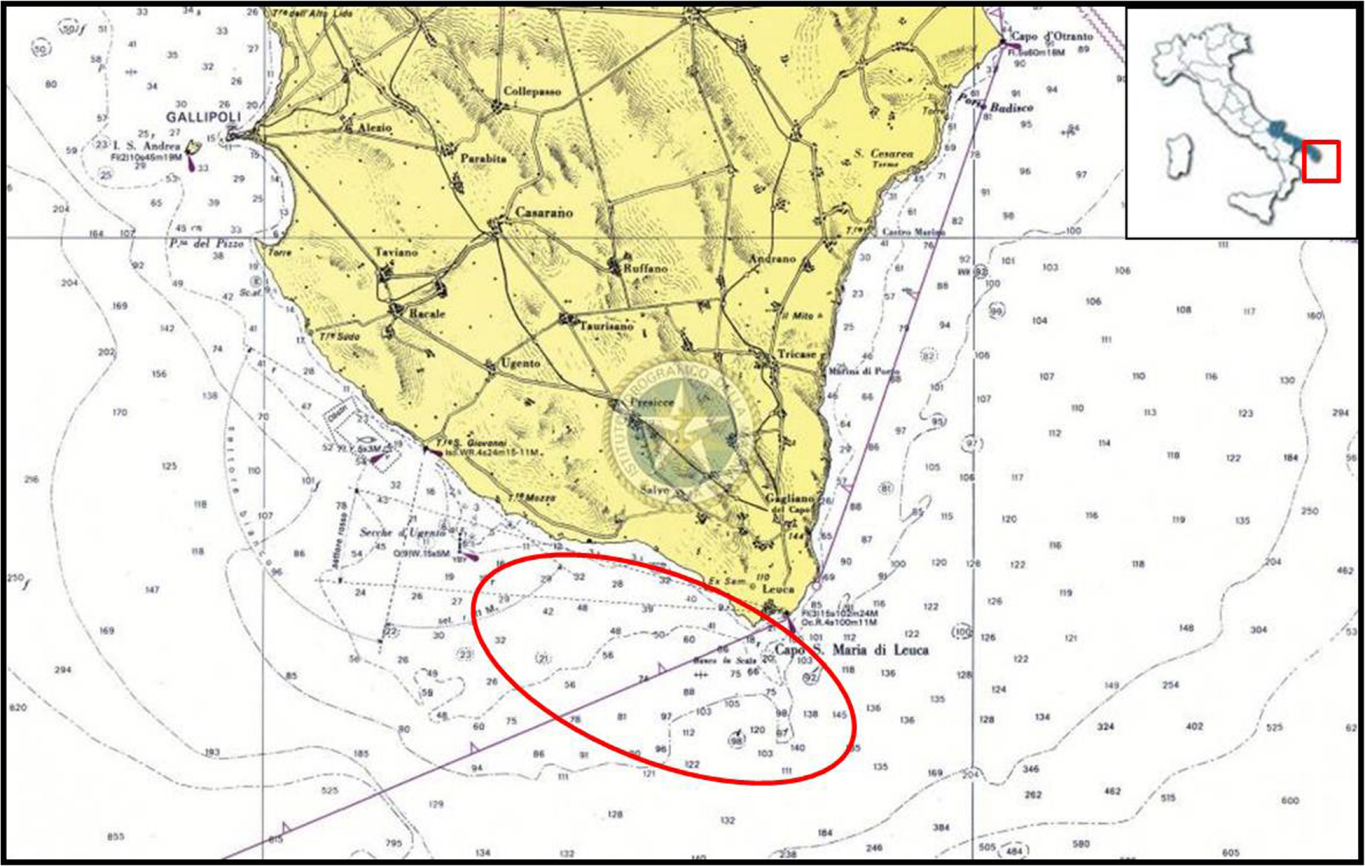
The red circle indicates the sampling area where moribund fish were collected, in the marine area surrounding the municipality of Santa Maria di Leuca (Northern Ionian Sea, Italy).

**Figure 2:**
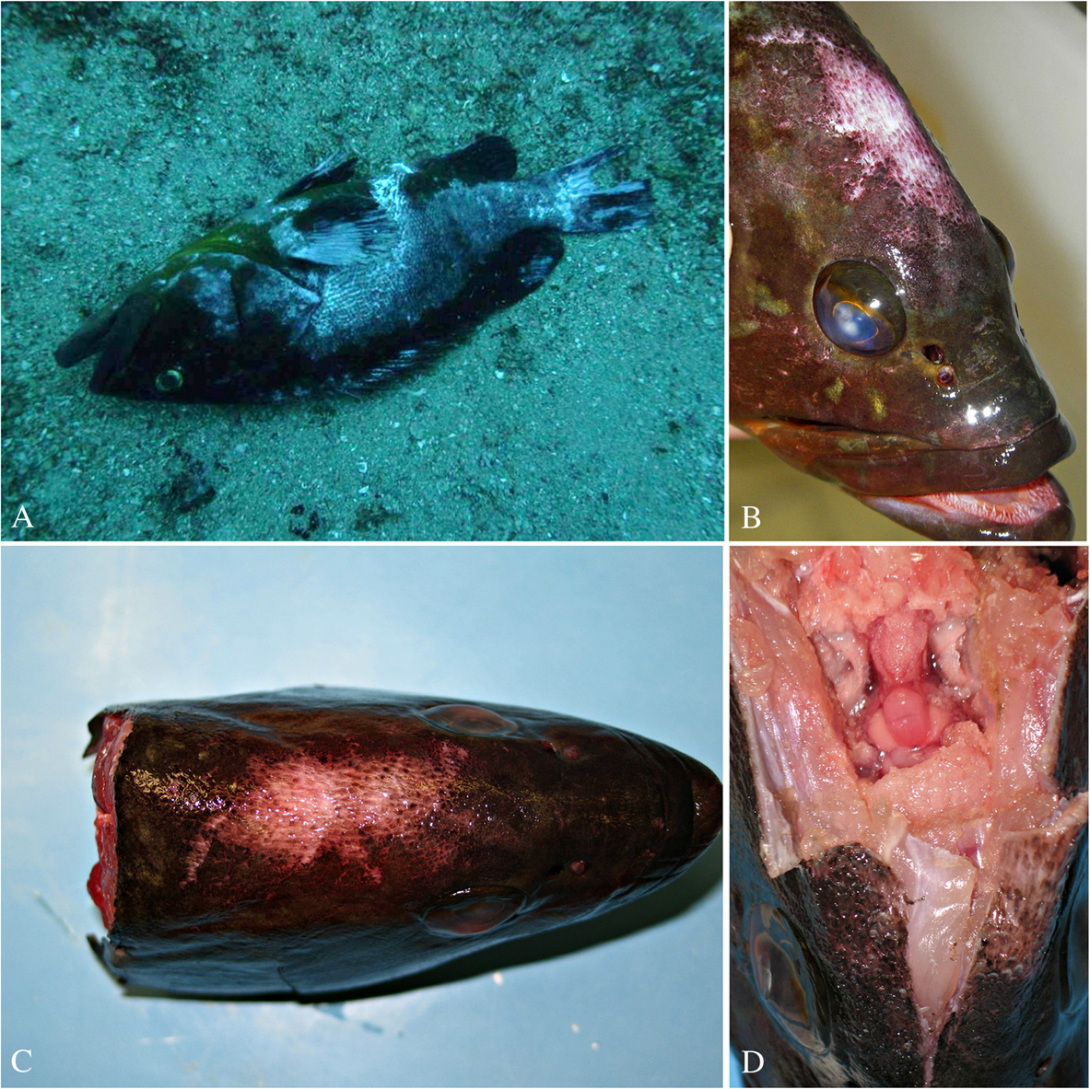
**VER infected dusky grouper. A**) Dead dusky grouper laying on the sea bottom, showing erosions of the fin likely associated with loss of swimming control and repeated trauma against rocks. **B** and **C**) Dusky grouper displaying head skin erosion and corneal opacity **D**) CNS hyperaemia in the same grouper of **B** and **C**.

In that period, fishermen reported the presence of a high number of dead or moribund wild fish (>200 specimens) in the area of sea surrounding the municipality of Santa Maria di Leuca, (Northern Ionian Sea, Italy (Figure 1). At the same time, scuba divers related the occurrence of dead groupers in rock caves and, notably, videotaped a moribund specimen characterized by an abnormal swimming behaviour (Figure 2; Additional file 1). The occurrence of mortality in wild fish was widely discussed by local media and reported as being an unusual phenomenon.

Five clinically affected wild adult fish, belonging to three different species, were caught by local veterinary authorities and submitted to the laboratory for analysis, namely *n*=3 golden grouper (*Epinephelus costae*), *n*=1 dusky grouper (*E. marginatus*) and *n*=1 European sea bass (*Dicentrarchus labrax*). At the time of capture, all fish displayed an abnormal swimming behaviour, loss of swim bladder control, blindness and skin erosion in the head region. Necropsy and bacteriological analysis on tissue specimens from kidney, spleen and central nervous system (CNS) were carried out according to standard procedures. Spleen, kidney and CNS from diseased fish were taken with sterile loop and plated onto Blood Agar medium. The plates were incubated at room temperature for 48 h and checked daily.

Virological investigations were performed by means of Real time PCR [17] and virus isolation [18] on a total of 17 samples collected from the central nervous system (CNS), and, whether available, retina, anterior kidney, blood and gonads (Table 1). According to Panzarin et al. 2012 [4], for each specimen sequencing and phylogenetic analysis were performed on viral strains isolated from the brain, with the exception of sample 425.1 isolated from the retina. Using the MEGA 4 package [19], sequences from both genes (867 bp-long RNA1 fragment and 504 bp-long RNA2 fragment) were aligned and compared to sequences publicly available in GenBank, as well as with unpublished sequences of viruses isolated from fish reared in sea cage farms during previous outbreaks of VER in the same area (Table 2). Phylogenetic trees were developed for both genetic segments using the neighbour-joining (NJ) method with 1000 bootstrap re-samplings. Nucleotide similarities were also determined.

**Table 1:** Samples collected from clinically affected golden grouper, dusky grouper and European sea bass, and molecular and virological analytical results

**Samples ID**	**Host species**	**Sample matrix**	**Real Time PCR (CP)**	**Cell culture isolation**
385.1	*E. marginatus*	CNS	+ (9.48)	+
385.2		Retina	+ (15.23)	+
385.3		Anterior kidney	+ (28.43)	+
386.3		Blood	-	-
396.3	*E. costae*	CNS	+ (17.59)	+
396.4		Optical nerve	+ (26.93)	+
424.1	*D. labrax*	CNS	+ (14.34)	+
424.2		Retina	+ (15.06)	+
424.3		Optical nerve	+ (21.69)	+
424.4		Anterior kidney	+ (28.99)	-
424.5		Gonads	-	-
425.1	*E. costae*	Optical nerve	+ (32.50)	+
425.2		Retina	+ (15.33)	+
425.3		Anterior kidney	+ (19.37)	+
496.1	*E. costae*	CNS	+ (16.8)	+
496.2		Retina	+ (14.5)	+
496.3		Anterior kidney	+ (25.3)	-

**Table 2:** Data collected in this study on isolates included in the phylogenetic analysis and related GenBank accession number

**Strain**	**Year**	**Host**	**Clinical sings**	**Status**	**GenBank accession no.**	
					**RNA1**	**RNA2**
332.2	2006	*D. labrax*	Not available	Farmed	JN189872	JN190012
327.7	2008	*D. labrax*	Present	Farmed	JX290528	JX290538
352.1	2008	*D. labrax*	Present	Farmed	JX290526	JX290540
549.13	2008	*D. labrax*	Present	Farmed	JX290527	JX290536
2.5	2009	*D. labrax*	Present	Farmed	JX290524	JX290537
2.6	2009	*D. labrax*	Present	Farmed	JX290525	JX290539
469.1	2010	*D. labrax*	Present	Farmed	JX290529	JX290541
469.2	2010	*D. labrax*	Present	Farmed	JX290530	JX290542
385.1	2011	*E. marginatus*	Present	Wild	JX290520	JX290532
396.3	2011	*E. costae*	Present	Wild	JX290521	JX290533
424.1	2011	*D. labrax*	Present	Wild	JX290522	JX290534
425.2	2011	*E. costae*	Present	Wild	JX290523	JX290535
496.1	2011	*E. costae*	Present	Wild	JX290531	JX290519

## Results and discussion

VER has been reported in the Mediterranean basin since 1991 and has proved to be a major problem for reared marine fish [11,15,20,21]. Nevertheless, subsequent epidemiological investigations highlighted the presence of betanodavirus also in several apparently healthy wild species [5,7,14]. Some sporadic cases of mortality in wild groupers likely associated to VER were reported in the Mediterranean basin, but so far only few laboratory-confirmed cases have been described [13,22]. Currently, detailed information on the occurrence of the disease in wild stocks in this area is missing.

All five fish collected for primary necropsy and further focused laboratory investigation showed: erosions, ulcers and scale loss in the head region, swim bladder hyperinflation, corneal opacity; hyperhaemia with spread blood vessel congestion of CNS was also observed (Figure 2).

Bacteriological examinations for the detection of common systemic bacterial pathogens yielded negative results. Virological analysis revealed that 15 out of 17 samples tested positive for betanodavirus detection by real time PCR, 13 of which were confirmed by virus isolation in cell culture (Table 1).

**Figure 3:**
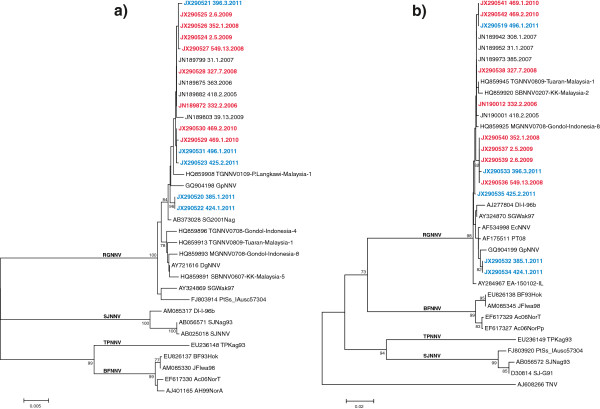
**Neighbour-joining (NJ) phylogenetic trees.** (**a**) partial RNA1-based phylogeny. (**b**) partial RNA2-based phylogeny. Strains isolated from wild diseased golden grouper, dusky grouper and European sea bass are labelled in blue, while betanodaviruses detected in farmed European sea bass reared in close proximity of the epidemic area are labelled in red. The numbers at branch points represent bootstrap values expressed as percentages (only values ≥ 70 are reported). The genotype subdivision according to Nishizawa et al., [3] is shown at the main branches. Scale bar represents nucleotide substitutions per site.

The phylogenetic trees inferred for the polymerase (RNA1) and the coat protein (RNA2) partial genes consistently grouped all the isolates within the RGNNV genotype, the most common in the Mediterranean sea (Figure 3). Sequences related to strains isolated from golden grouper (396.3.2011, 425.2.2011, 496.1.2011) showed high nucleotide similarity (99.8-100% for RNA1; 99.4-100% for RNA2) with a panel of betanodavirus strains isolated during clinical outbreaks occurred in previous years (2006–2009) in European sea bass reared in neighbouring fish farms. Lower nucleotide similarity (99.7-99.8% for RNA1 and 99.1-99% for RNA2) was observed between sequences related to viral strains detected in farmed fish and samples 385.1.2011 and 424.1.2011. Interestingly, viral strains isolated from dusky grouper and European sea bass (samples 385.1 and 424.1, respectively) were identical and phylogenetically distinguishable from the golden grouper betanodaviruses. Overall, similarity ranges among betanodavirus strains herein characterized were 100–99.7% and 100-99% for RNA1 and RNA2, respectively.

The mortality rate of an outbreak in wild fish is generally extremely difficult to evaluate since several variables which are quite difficult – if not impossible – to measure should be considered. For instance, it is very hard to assess the extent of the outbreak if a great number of dead fish inside rock caves in the area is found, or in case moribund fish on the sea surface (due to swim bladder inflation) are collected by fishermen or captured by other fish or birds.

However, the estimated number of dead fish (more than 200) reported by local authorities, fishermen and diving centres is an indication of how severely this outbreak affected wild stocks.

The high water temperature has often proved to be an environmental factor associated with the development of clinical disease within aquaculture activities [23]. Thus, given the thermal anomalies characterising the water column during the outbreak, the onset of clinical disease in wild populations can be ascribed to this condition.

Aquaculture facilities, consisting in 12 floating cages, are located approximately 15–20 kilometres North-East from the place of the epidemic. In these cages European sea bass (*D. labrax*) Gilthead sea bream (*Sparus aurata*) and some other minor species are reared and fattened for human consumption with a production of 400 tons per year. Considering that data on the health condition of the farmed fish during the outbreak in the wild species herein described were not available, viral sequences belonging to strains isolated in the area from previous years in farmed fish were included in the phylogenetic study.

To date, only few data are available regarding viral transmission between marine wild and farmed fish [4] and it is still unknown which is the direction of the viral flow (i.e. wild to farmed fish or viceversa) -possibly the flow is bidirectional- [22]. Regarding this specific case, the high genome similarity among viruses isolated in different years in wild and farmed fish from the same area is highly suggestive of a persistent circulation of betanodaviruses and transmission between wild and farmed stocks. Due to a lack of monitoring plans in wild fish populating that area, further studies are required to assess the risk of spreading pathogen(s) from/to the wild.

## Conclusions

In the present study we described a severe outbreak of VER in three different wild carnivore fish species, namely dusky grouper, golden grouper and European sea bass in the Northern Ionian Sea, Italy. To the authors’ knowledge, this is the first documented report describing a disease outbreak in wild species in the Mediterranean basin, with a documented description of clinical signs associated to the isolation and characterization of the causative agent. These results are consistent with the report by Gomez and colleagues [24] describing for the first time an outbreak of VER in wild adult *E. akaara* in Japan, and confirm the high susceptibility of the genus *Epinephelus* to this pathogen.

The observation of this outbreak possibly represents a starting point for further investigations aiming to evaluate pathogens transmission between farmed and wild fish and to assess the risk of viral exchange between wild and farmed fish populations. The potential transmission of the disease from farm to wild or vice versa must be seriously taken in consideration, with particular reference to the management of marine protected areas (MPA) and fish preservation projects.

## Abbreviations

VER: Viral encephalopathy and retinopathy; CNS: Central nervous system.

## Competing interest

The authors declare that they have no competing interest.

## Authors’ contributions

NV wrote the paper; PP designed and coordinated the study and together with PT and AT collected samples from the field and information about the mortality event of fish; EC and VP performed virological and phylogenetic analysis respectively; AT supervised the laboratory activities and helped to draft the manuscript; CT and GC have given final approval of the version to be published. All authors read and approved the final manuscript.

## Supplementary Material

Additional file 1 **Dusky grouper (*E. marginatus*) moribund specimen video-recorded in October 2011 from a scuba diving instructor in Santa Maria di Leuca.** This fish, observed 30 mt deep, displays an abnormal swimming behaviour and blindness. Samples 385.1 (CNS), 385.2 (retina), 385.3 (anterior kidney) and 386.3 (blood) were collected from this specimen and subjected to laboratory investigations. Betanodavirus was detected by Real Time PCR and viral isolation in cell culture (SSN-1) in all the samples, with the exception of sample 386.3 (blood) which yielded negative results. Click here for file
